# P-1590. Diagnostic Stewardship as a CAUTI Prevention Strategy: Implementation of a Urine Culture Wizard to Drive Institutional Change

**DOI:** 10.1093/ofid/ofae631.1757

**Published:** 2025-01-29

**Authors:** Claire Reisenauer, Alyssa Y Castillo, Shane Hansen, Jennifer Bovaird, Keri Moylan, Emily Robbins, Larissa Pisney

**Affiliations:** University of Colorado Hospital, Aurora, Colorado; University of Colorado Hospital, Aurora, Colorado; UCHealth EPIC Inpatient, Aurora, Colorado; UCHealth, Aurora, Colorado; University of Colorado Hospital, Aurora, Colorado; University of Colorado Hospital, Aurora, Colorado; University of Colorado School of Medicine, Aurora, Colorado

## Abstract

**Background:**

Standardized urine culture ordering is an essential practice in the prevention of catheter-associated urinary tract infections (CAUTI). Historically, the University of Colorado Hospital (UCH) offered a stand-alone urine culture order, which required documentation of the culture indication. Frequently, the attestation was incongruent with the clinical scenario, and a preceding urinalysis (UA) was not performed. As a result, cultures often resulted in the detection of asymptomatic bacteriuria, leading to inappropriate antibiotic prescribing. We hypothesized that the creation of a Urine Culture Wizard would decrease unnecessary urine cultures with downstream implications on antibiotic usage and CAUTI reporting.

Year-Over-Year % Change by Month and Fiscal Year

**Figure 1:**
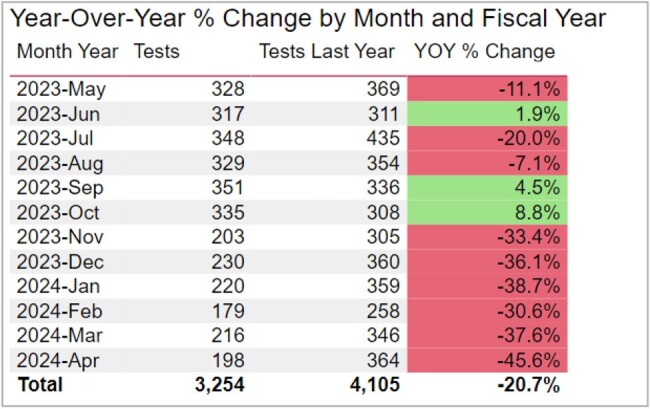
Year-over-year Reduction in Urine Cultures Performed at UCH by Inpatient Teams

**Methods:**

In 2021, key stakeholders across the UCHealth system began the development of a Urine Culture Wizard. This replaced all orders for urine cultures in inpatient settings. Multiple synonyms push providers to the wizard, which performs data mining to determine if a urine culture is appropriate. For most, it requires that a preceding UA showing pyuria has been performed. The wizard also allows the ordering of a urine culture without documentation of pyuria if unique patient characteristics are detected in the electronic medical record. These are identified by discrete fields that correspond to criteria as outlined by an institutional governance committee, including specific clinical diagnoses. Educational material was developed to support the launch of the wizard.

Year-Over-Year % Change by Month and Fiscal Year

**Figure 2:**
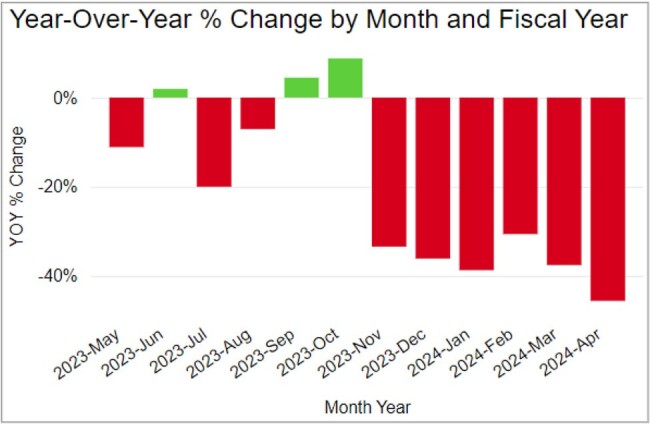
Year-over year Reduction in Urine Cultures Performed at UCH by Inpatient Teams

**Results:**

The Urine Culture Wizard went live in November 2023. Results show a sustained decrease of more than 30% in the number of urine cultures ordered at UCH, and this change meets the criteria for special cause variation. (Figures 1, 2, and 3).

Urine Tests Per Month

**Figure 3:**
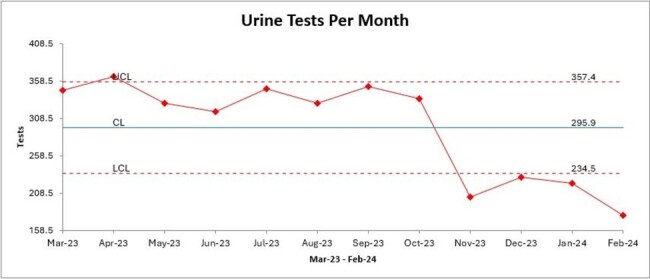
Statistical Process Control Chart Demonstrating Special Cause Variation

**Conclusion:**

It has been shown that inappropriate ordering of urine cultures leads to misclassification of colonization as infection and unnecessary prescribing of antibiotics. A standardized approach to ordering urine cultures based on clinical criteria was created for all UCHealth hospitals through the development of a Urine Culture Wizard. Since implementation there has been a continual significant reduction in the number of urine cultures performed, signaling the success of this diagnostic stewardship tool.

**Disclosures:**

**All Authors**: No reported disclosures

